# Stigma and quality of life in hospitalized schizophrenia patient-family caregiver dyads in Northern China: an actor-partner interdependence model analysis

**DOI:** 10.3389/fpsyt.2026.1855525

**Published:** 2026-06-30

**Authors:** Kang Xie, Honglei Yang, Xinyi Ge, Qunshan Wang, Zhenyue Liu, Xianyun Li, Botao Ma

**Affiliations:** 1 Graduate School, Chengde Medical University, Chengde, China; 2 Third Clinical Department, Beijing Huilongguan Hospital, Capital Medical University, Beijing, China; 3 Beijing Fengtai District Mental Health Center, Beijing, China

**Keywords:** actor-partner interdependence model, family caregiver, quality of life, schizophrenia, stigma

## Abstract

**Background:**

Schizophrenia is a chronic and relapsing mental disorder that is consistently associated with a severely diminished quality of life (QoL) for patients. Existing research has predominantly focused on how the stigma experienced by patients with schizophrenia relates to their own QoL. However, stigma among family caregivers has received considerably less attention, and its potential association with patients’ QoL, in particular, remains underexplored. Therefore, this study aims to systematically analyze the dyadic associations of stigma—as experienced by both patients with schizophrenia and their family caregivers—with QoL, utilizing an actor-partner interdependence model (APIM). Through this framework, this study seeks to explore the interdependence of stigma between patients and their family caregivers and its correlational links to their quality of life.

**Methods:**

Two hundred and sixty-four pairs of schizophrenic patients and their family caregivers were included, and the subjects’ stigma was measured using the Internalized Stigma of Mental Illness Scale and the Conjunctive Stigma Scale, respectively, and the quality of life was measured using the World Health Organization Quality of Life Measurement Short Form. The actor-partner effect of stigma on quality of life was explored by constructing an actor-partner reciprocity model.

**Results:**

The actor effect of stigma on quality of life was significant for people with schizophrenia and their family caregivers (β=-0.472, *p* < 0.001, β=-0.779, *p* < 0.001), and the partner effect of stigma on quality of life was significant for people with schizophrenia and their family caregivers (β=-0.128, *p* = 0.033, β=-0.419, *p* < 0.001).

**Conclusion:**

In future research and interventions aimed at improving the quality of life for people with schizophrenia and their caregivers, it is important to consider not only the individual’s own stigma, but also how the other person’s stigma is associated with one’s quality of life.

## 1 Introduction

Schizophrenia is a prevalent and severely disabling mental disorder that affects millions of individuals and their families globally (1, 2), with patients often experiencing symptoms such as hallucinations and delusions, which severely affect their social functioning and thus inevitably have a profoundly negative impact on their QoL (3). However, the impact of schizophrenia goes far beyond the individual sufferer; it also places a heavy burden on the entire family system, especially the family caregivers who have the primary caregiving responsibilities (4). Family caregivers must continually confront patients’ fluctuating symptoms, manage practical difficulties in daily life, and endure significant emotional distress and potential social prejudice. These challenges also severely impact the caregivers’ own QoL (5). Therefore identifying and understanding the key factors affecting the quality of life of patients and their family caregivers is critical to the overall management of the disease.

Stigma is one of the key factors affecting patients’ recovery and QoL (6). Stigma can be categorized into public stigma, self-stigma, and associative stigma (7). On the one hand, patients with schizophrenia suffering from self-morbid stigma has been shown to be significantly associated with lower self-esteem, depressed mood, and decreased QoL (7). At the same time, family caregivers experience associative stigma as a result of their association with the patient, and this stigma also negatively affects their QoL (8).

Consequently, clarifying the interplay of stigma and QoL within patient-family caregiver dyads is vital for understanding their adaptive status. To achieve this, the Actor-Partner Interdependence Model (APIM) serves as a robust analytical framework designed specifically for dyadic data (9). The APIM allows for the concurrent estimation of actor effects (the association between an individual’s characteristics and their own outcomes) and partner effects (the cross-partner association between an individual’s characteristics and their partner’s outcomes). In this study, we evaluated actor effects (the associations of patients’ stigma and family caregivers’ affiliate stigma with their respective QoL) and, crucially, partner effects (the cross-partner associations between these stigma constructs and the other’s QoL).

Prior research using the Actor-Partner Interdependence Model (APIM) has explored the relationships between stigma and QoL within these dyads. For instance, a study of outpatients in Southern China revealed a significant partner effect only from caregivers to patients (where the caregiver’s stigmatizing attitude was significantly associated with patient QoL), but found no corresponding partner effect from patients to caregivers, meaning patient self-stigma was not significantly associated with caregiver QoL (10).However, in traditional Chinese culture—characterized by filial piety and deep familial interdependence—patients and their caregivers often form a ‘community of shared honor and disgrace’ (11). Within this collectivistic framework, the negative consequences of a patient’s self-stigma are highly likely to be closely linked to the caregiver’s well-being and QoL (8). thus, the relationship between patient stigma and caregiver QoL warrants further investigation. Furthermore, the aforementioned study presents certain methodological limitations. First, its sample was restricted to outpatients from Southern China, limiting its representativeness. Second, the measurement of caregiver stigma relied on a modified patient self-report scale, which may have failed to precisely capture the unique construct of affiliate stigma—the specific form of stigma experienced by caregivers due to their association with the patient.

To address these limitations, the present study focuses on a previously unexamined population—hospitalized individuals with schizophrenia and their family caregivers in Northern China—and employs the validated Affiliate Stigma Scale to ensure precise measurement. The specific aims of this study are (1): to re-examine the actor and partner effects (modeled as the associations of patient self-stigma and family caregiver affiliate stigma with their own and partners’ QoL) within this distinct clinical population and cultural context, with a particular focus on the cross-partner association between patient stigma and caregiver QoL; and (2) to provide preliminary data that may inform the development of culturally-tailored, dyadic intervention strategies designed to support both patients and their family caregivers in the Chinese context.

## 2 Method

### 2.1 Participants

This is a cross-sectional study, using convenience sampling method to investigate schizophrenia patients and their primary caregivers who were hospitalized in Beijing Huilongguan Hospital between November 2023 and January 2025. Inclusion criteria for patients with schizophrenia (1): meeting the diagnostic criteria for schizophrenia in the Diagnostic and Statistical Manual of Mental Disorders, 5th edition (DSM-5) (2); between the ages of 18 and 65 years old (3); having a middle school or higher Cultural level, no Chinese reading and writing disabilities, and able to cooperate with the completion of various scales. Exclusion Criteria for patients (1): having impaired consciousness or serious organic diseases (2); having reading comprehension or communication disorders (3); having serious cognitive deficits (4); having low intelligence (5); having other mental disorders or personality disorders (6); having a history of substance abuse, such as drugs or alcohol (except tobacco).

Inclusion criteria for primary family caregivers: 1) Aged 18 years or older; 2) The patient’s legal guardian, such as a spouse, parent, adult child, or sibling; 3) Identified as the family member with primary responsibility for the patient’s daily life, treatment, and emotional support for at least the past 6 months; 4) Having a junior high school education level or above, with no impairments in reading or writing Chinese, and able to cooperate in completing all survey scales. Exclusion criteria for family caregivers: 1) Presence of a severe mental illness; 2) Presence of a severe physical illness or cognitive impairment; 3) Having a low level of intellectual functioning; 4) A history of drug, alcohol, or other substance abuse (excluding tobacco).

In this study, the cross-sectional survey sample size was determined using the cross-sectional survey sample size formula *n*=(μ_α_ σ/δ)^2^, where the allowable error was set at δ=2 and α=0.05. Taking into account a sample dropout rate of approximately 20%, it was calculated that at least 170 pairs would be needed, but structural equation modeling requires a sample size of at least 200 pairs or more, and it was expected that 300 pairs of people with schizophrenia and their caregivers would be included.

Initially, a baseline sample size of 170 pairs was calculated using the formula *n*=(μ_α_ σ/δ)^2^ (with δ=2, α=0.05, and an anticipated 20% attrition rate). However, to provide sufficient statistical power for the Actor-Partner Interdependence Model (APIM) analyzed via structural equation modeling, we ultimately recruited and analyzed 300 valid patient-caregiver dyads. These 300 dyads (corresponding to 600 individuals) yield a sample-to-parameter ratio (N:q) of approximately 20:1 (or 40:1 if calculated based on the 600 individuals) for the approximately 15 estimated parameters, which fully satisfies the recommended 10:1 to 20:1 ratio for stable estimation in structural equation modeling.

### 2.2 Procedure

Prior to any data collection, the study was approved by the Ethics Committee of Beijing Huilongguan Hospital [2025-75-Science]. Data were subsequently collected by a team of three psychology graduate students and one psychiatrist. All collectors possessed over two years of clinical internship experience in psychiatric wards, ensuring they had the professional competence for effective communication with psychiatric patients. Furthermore, all researchers underwent standardized training specific to this research protocol, covering the study’s objectives, standardized instructions, the content of each scale, and standard procedures for handling potential issues during data collection.

All participants provided written informed consent. Researchers explained the purpose of the study in detail to both the patients with schizophrenia and their primary family caregivers before administering the questionnaires to those who agreed to participate. If both the patient and the family caregiver were present, they were asked to complete the questionnaires separately, and the completed forms were collected on-site. If the family caregiver was not present, they were asked to complete the survey during their next hospital visit. It was emphasized that both parties should not discuss their answers with each other and should respond based on their initial reactions. The survey was conducted in a quiet and private room to minimize distractions. Throughout the process, researchers monitored the participants’ condition, allowing for breaks if a patient showed signs of inattention. The entire survey took approximately 10-15 minutes to complete. Upon completion, participants received a small gift as a token of appreciation.

A total of 300 pairs of schizophrenia patients and their caregivers participated in this study. After excluding 36 pairs of data with severe missing values (e.g., >10% missing in core scales), invalid responses (e.g., patterned responses), or missing key information, 264 valid paired data sets were ultimately obtained for analysis, yielding an effectiveness rate of 88.0% (12, 13).

The data for the present study were drawn from a larger research project on the psychosocial well-being of schizophrenia dyads. We hereby clarify that this study uses the same population as in a previous publication by the authors in BMC Psychiatry. Despite the shared population, the current manuscript represents a significant and foundational progression from our prior work. The two studies employ different models to address scientific questions at two distinct levels: The previous study utilized a more complex Actor-Partner Interdependence Mediation Model (APIMeM) to investigate the mediating role of social support in the relationship between stigma and quality of life. Its core question was “How” the effect occurs, focusing on the underlying mechanism. In contrast, the present study focuses on a more fundamental and direct question. We applied the basic Actor-Partner Interdependence Model (APIM) to first establish and quantify the direct actor and partner effects between stigma and quality of life. Its core question is “If and How Much” an effect exists. The direct cross-person effects revealed in this study are foundational to understanding more complex mediation models and were not independently and thoroughly reported in the prior work. Therefore, the current manuscript is not a repetition, but rather a foundational and dedicated analysis of the direct effects of stigma in the dyadic context, providing both essential validation and an independent contribution complementary to the more complex mediation model in our previous research.

### 2.3 Measures

#### 2.3.1 General information and clinical data

A questionnaire was used to collect general and clinical information about the subjects, including their age, sex, residence, marital status, and education level. The Internalized Stigma Scale for Mental Illness, developed by Ritsher et al. in 2003 and validated by Chinese scholars, can be used to assess the internalized stigma of schizophrenia patients, with good reliability and validity.

#### 2.3.2 Internalized stigma of mental illness scale

The Internalized Stigma Scale for Mental Illness, developed by Ritsher et al. in 2003 and validated by Chinese scholars, can be used to assess the internalized stigma of schizophrenia patients, with good reliability and validity (14, 15). The scale consists of 29 entries. The scale consists of a detachment factor, a stereotypical identification factor, a social withdrawal factor, a discrimination experience factor, and a stigma resistance factor, and is scored on a Likert 4-point scale ranging from 1 (strongly disagree) to 4 (strongly agree). A mean score was calculated by dividing the total score by the number of items, with higher scores indicating a more severe level of patient stigma. In this study, a mean score > 2.0 was defined as the presence of stigma. The internal consistency coefficient of the scale was 0.922 (15).

#### 2.3.3 Affiliate Stigma Scale

The Affiliate Stigma Scale was developed by Hong Kong scholars Mak et al. (16), which is mainly used to assess the sense of guilt of family members of patients with mental illness. The scale consists of 22 entries, including 3 dimensions of cognitive, affective and behavioral responses, with a Likert 4-point scale ranging from 1 (strongly disagree) to 4 (strongly agree), the scale was scored using the mean of all items, where a higher score reflects a greater degree of patient stigma. A mean score above 2.0 was considered indicative of stigma and the scale has a total Cronbach’s α coefficient is 0.94, which is currently the most widely used scale to measure guilt by association of family members of patients with schizophrenia (17).The Internalized Stigma of Mental Illness (ISMI) Scale and the Affiliate Stigma Scale are commonly used instruments for measuring the stigma experiences of patients with schizophrenia and their family caregivers, respectively. Although they target different populations and specific manifestations of stigma, both scales are designed to quantify the core psychological construct of stigma (18).

#### 2.3.4 World Health Organization Quality of Life measurement scale

The World Health Organization Quality of Life Measurement scale is used to measure the quality of life of an individual and consists of 26 entries, including 2 overall assessments of total health status and quality of life and 4 dimensions: physical (7 entries), psychological (6 entries), social relationships (3 entries) and environmental (8 entries). A 5-point Likert scale ranging from 1 (very dissatisfied) to 5 (very satisfied) was used, and several dimensions were totaled into a scale total score, with higher scores representing better quality of life. It has good reliability and validity in the schizophrenia population, and the internal consistency coefficient of the scale is 0.847 (19).

### 2.4 Statistical methods

The collected data were analyzed using SPSS 26.0 and AMOS 24.0. Descriptive statistics and correlation analyses were performed using SPSS to analyze the general and clinical information about the subjects and the data measured by the people with schizophrenia and their caregivers, and to check whether the variables belonged to normally distributed data. The AMOS structural equation modeling was used to determine the actor-partner effect of stigma on the quality of life of people with schizophrenia and their caregivers because of its advantages of statistical comparison and model validation.

Absolute fit indices were used to assess the fit of the model, including the chi-square test (χ^2^), degrees of freedom (df), root mean square error of approximation (RMSEA), and goodness of fit index (GFI). Incremental fit indices included the comparative fit index (CFI), normed fit index (NFI), and incremental fit index (IFI) (20). Finally, the Bootstrap method in AMOS (set to 5,000 resamples) was used to estimate the standard errors and 95% bias-corrected confidence intervals to verify the statistical significance of the direct actor and partner paths in the model.

## 3 Results

### 3.1 Demographic characteristics of schizophrenic patients and their family caregivers

As shown in **Table 1**, among the patients with schizophrenia, the gender distribution was relatively balanced (52.3% male), and the average age was approximately 45.54 years. The majority of patients (58.00%) were unmarried, and over half (52.3%) had attained a junior college level of education or higher. For the family caregivers, the average age was approximately 52.60 years, and the vast majority (89.01%) were married. The primary caregiving relationships were siblings (40.15%) and parents (36.36%).

**Table 1:** Demographic characteristics of people with schizophrenia and their caregivers (n=264 pairs).

Variables	Classify	*Group*/*n*(%)		
		Patients with schizophrenia		Family caregiver
Gender	Male	138 (52.27%)		150(56.82%)
	Women	126 (47.73%)		114 (43.18%)
Age	18-30	42 (15.91%)		12 (4.55%)
	31-40	52 (19.70%)		22 (8.33%)
	41-50	46 (17.42%)		77 (29.17%)
	51-65	124 (46.97%)		153 (57.95%)
	Mean ± Standard deviation	45.54 ± 12.63		52.60 ± 11.27
Educational level	Junior middle school	56 (21.21%)		64 (24.24%)
	Junior high school	70 (26.52%)		74 (28.03%)
	Junior college education	43 (16.29%)		58 (21.97%)
	Undergraduate	82 (31.06%)		53 (20.08%)
	Postgraduate	13 (4.92%)		15 (5.68%)
Family registry	Urban	209 (70.83%)		208 (78.79%)
	Rural	55 (29.17%)		56 (21.21%)
Caregiver's relationship to the patient	Father and mother	96 (36.36%)		
	Spouse	43 (16.29%)		
	Sons and daughters	19 (7.20%)		
	Siblings	106 (40.15%)		
Marital status	Single	153 (57.95%)	23 (8.71%)	
	Married	64 (24.24%)	235 (89.02%)	
	Widowed	2 (0.76%)	2 (0.76%)	
	Divorcee	45 (17.05%)	4 (1.51%)	

### 3.2 Differential and correlational analysis of stigma and quality of life in patients and caregivers with family schizophrenia

As shown in **Table 2**, the quality of life of schizophrenic patients was lower than that of their family caregivers (79.92*vs.*83.13, *p*<0.001), whereas there was no significant difference between the two for the other scale scores. The results of Pearson’s correlation analysis showed that the total score of the schizophrenic patient’s stigma was significantly correlated with his/her own quality of life, and with the total score of the family caregiver’s stigma and quality of life (*r* = -0.84, 0.89, -0.82, *p*<0.001). Family caregivers’ affiliate stigma scores were negatively and significantly correlated with both the patient’s and their own quality of life (*r* = -0.84, -0.89, *p*<0.001). The total quality of life score of family caregivers of patients with schizophrenia was significantly and positively correlated with the total quality of life score of family caregivers (*r* = 0.94, *p*<0.001).Given that patient self-stigma and family caregiver’s affiliate stigma were measured using different scales, the comparison of their mean scores is intended solely for descriptive purposes and should be interpreted with caution.

**Table 2:** Means, standard deviations, t-values and correlation matrix for each variable (n=264 pairs).

	M	SD	*t*	A	B	C	D
A	2.19	0.45	-1.93	1			
B	2.23	0.57		0.89^***^	1		
C	79.92	22.39	-6.53^***^	-0.84^***^	-0.84^***^	1	
D	83.13	23.68		-0.82^***^	-0.89^***^	0.94^***^	1

A = Patient stigma, B = Family caregiver stigma, C= Patient quality of life, D= Family caregiver quality of life. t-values represent paired-samples t-test comparisons between patient and caregiver scores (i.e., A vs. B, and C vs. D)***, p < 0.001.

Given the significant correlations among the study variables, we formally evaluated potential multicollinearity prior to constructing the structural equation model. Multicollinearity diagnostics were performed using Variance Inflation Factors (VIF) and tolerance values. Results indicated that the VIF values for all predictor variables ranged from 4.88 to 7.84, with tolerance values ranging from 0.13 to 0.21, none of which exceeded the widely accepted threshold of VIF < 10 and tolerance > 0.10. Notably, the VIF for the family caregiver’s QoL was 7.84, which is slightly above the stricter threshold of 5.0. This elevation is theoretically expected in dyadic data, stemming from the inherent interdependence between actor and partner variables (i.e., the close correlation between patient and caregiver QoL). In sum, no severe multicollinearity issues were detected, supporting the robustness of the subsequent path analysis.

### 3.3 Test of measurement model

Based on the theoretical framework of the Actor-Partner Interdependence Model (APIM), a structural equation model was constructed to examine the relationships of patient self-stigma and family caregiver affiliate stigma with their own and partners’ quality of life (QoL) scores, with all variables treated as observed variables. The chi-square difference test showed a marginal difference between the constrained and unconstrained models (χ² = 3.940, p=0.139<0.2), indicating that the actor and partner effects were not equal across patients and caregivers (20). Consequently, the final model obtained in this study was a saturated (just-identified) model with zero degrees of freedom (df=0) and a chi-square value of zero (χ²=0). Therefore, all global fit indices, including the Comparative Fit Index (CFI), Goodness-of-Fit Index (GFI), Normed Fit Index (NFI), and Incremental Fit Index (IFI), were mathematically predetermined to equal 1.0 (21). Because traditional fit indices cannot evaluate the adequacy of a saturated model, model evaluation was primarily based on the significance and directionality of the path coefficients.

#### 3.3.1 An actor-partner interdependence model of stigma and quality of life in schizophrenia patient-caregiver dyads

**Figure 1:**
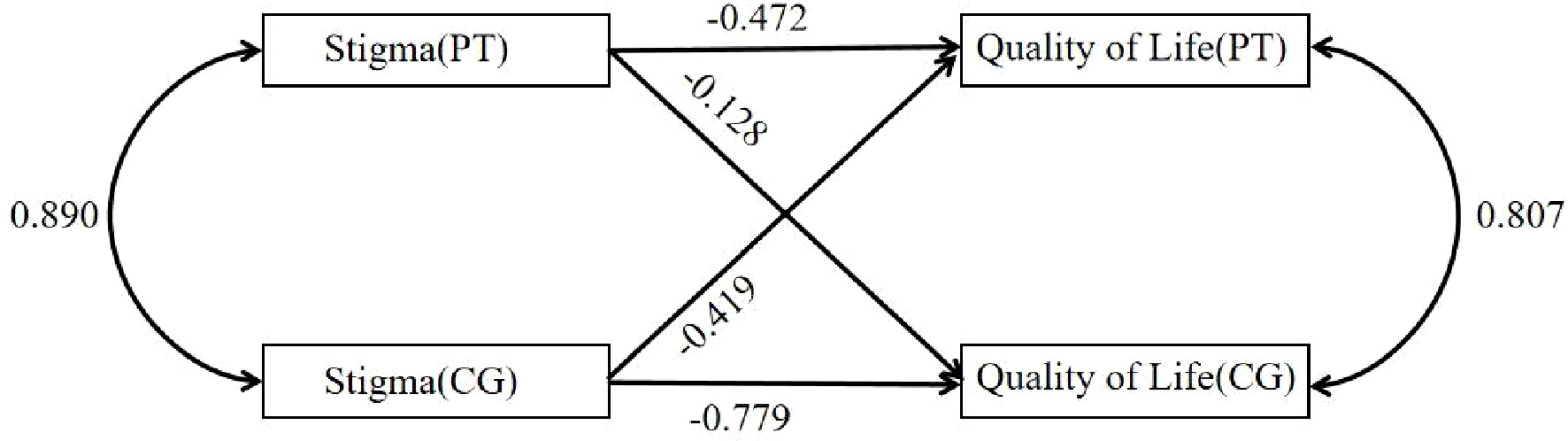
The Actor-Partner Interdependence Model of stigma and quality of life in schizophrenia patient-caregiver dyads. CG, caregiver; PT, patient; Values on the paths represent standardized path coefficients (*β*).

The results of the Actor-Partner Interdependence Model for the effects of stigma on QoL in patients with schizophrenia and their family caregivers are presented in **Table 3** and **Figure 1**. Regarding the direct effects, significant actor effects were found, indicating that a patient’s own stigma significantly predicted their own lower QoL (β=-0.47, *p* < 0.001), and a caregiver’s own stigma significantly predicted their own lower QoL (β = -0.78, *p* < 0.001). In terms of partner effects, both were also significant, revealing that the patient’s stigma predicted a lower QoL for the caregiver (β=-0.13, *p* = 0.033), while the caregiver’s stigma predicted a lower QoL for the patient (β=-0.42, *p* < 0.001).

**Table 3:** Effect coefficients for hypothetical model(n=264 pairs).

Trails	*coef*	*t*	*p*
Patient Stigma - Patient Quality of Life	-0.47	-6.98	<0.001
Patient Stigma - Family Caregiver Quality of Life	-0.13	-2.13	0.033
Family caregiver stigma-patient quality of life	-0.42	-6.19	<0.001
Family Caregiver Stigma - Family Caregiver Quality of Life	-0.78	-12.91	<0.001

## 4 Discussion

Utilizing the Actor-Partner Interdependence Model, this study investigated the impact of stigma on the QoL of patients with schizophrenia and their family caregivers. The findings demonstrate that stigma not only negatively affects an individual’s own QoL (an actor effect) but also significantly impacts their partner’s QoL (a partner effect), and these effects were reciprocal. These results underscore the necessity of concurrently addressing stigma in both patients and caregivers and highlight the need for comprehensive, dyad-based intervention strategies in schizophrenia care (22).

### 4.1 Current status of stigma and quality of life of patients and family caregivers with schizophrenia

In line with a substantial body of literature, our findings are consistent with the high prevalence of stigma among both patients with schizophrenia and their family caregivers (23, 24). Despite increased public awareness of mental illness, cultural traditions and social discrimination remain prevalent (25). On the patient’s side, the manifest symptoms of schizophrenia, both positive (e.g., hallucinations) and negative (e.g., emotional blunting, social withdrawal), are often accompanied by a sense of alienation and social ostracism. The internalization of these negative societal labels is frequently associated with profound feelings of shame, inferiority, and worthlessness (7, 26). Simultaneously, family caregivers experience a form of affiliate stigma, fearing being labeled as an “abnormal family” by association. This stigma is often linked to challenges in their social and professional lives and correlates with personal experiences of societal pressure, discrimination, and rejection (27).

Consistent with the findings of previous literature, this study found that the QoL of patients with schizophrenia was significantly lower than that of their family caregivers, a disparity that highlights the multifaceted burden associated with mental illness for patients (28). A potential explanation for this difference may be that patients directly experience the illness, whereas family caregivers navigate it indirectly. The full spectrum of schizophrenic symptoms (e.g., hallucinations, delusions, cognitive impairments, emotional blunting, social withdrawal) is intrinsically linked to the patients themselves, often corresponding with challenges in perception, thought, emotion, and behavior. The presence of these symptoms is strongly correlated with difficulties in daily life, making it challenging for them to live as healthy individuals do (29). In contrast, although family caregivers also report immense pressure and psychological burden, their lower QoL appears more related to the indirect aspects of the illness, such as caregiving responsibilities, financial strain, social isolation, and emotional distress (30, 31). While caregiving responsibilities consume significant time and energy, which may correlate with limitations in career development and social activities, most family caregivers are still able to maintain basic occupational and social functioning and often have access to more social resources to cope with life’s challenges (32). At its core, this study utilizes the Actor-Partner Interdependence Model (APIM) to examine the dyadic associations of stigma between patients and family caregivers and how these are related to their respective quality of life.

### 4.2 Correlation between stigma and quality of life in patients and family caregivers with schizophrenia

The correlational findings of this study highlight a strong pattern of interdependence within the dyad. Patient self-stigma was inversely associated not only with their own QoL but also with the QoL of their family caregivers, while being positively correlated with caregiver affiliate stigma. Rather than indicating a causal sequence, these cross-sectional data consistently suggest an intertwined negative associative pattern. Specifically, heightened patient self-stigma is concurrently associated with an increased psychological burden on their family caregivers and a lower caregiver quality of life (33). Similarly, the affiliate stigma reported by family caregivers is negatively correlated not only with their own QoL but also with that of the patients they care for, further underscoring the shared significance of caregiver stigma within the dyad. One potential context for these associations is that higher levels of caregiver affiliate stigma may be related to stress or negative attitudes within the family environment, which could align with the lower QoL observed in patients (33, 34). The positive correlation between patient and caregiver QoL further solidifies this notion of dyadic interdependence. This shared variance suggests that the dyad functions as an integrated unit, lending support to the hypothesis for future longitudinal studies that interventions tailored to support one member might be associated with positive outcomes for the dyad as a whole. Taken together, stigma constructs emerge as prominent correlates—rather than direct determinants—of QoL in these dyads, characterized by a complex pattern of mutual associations (35).

### 4.3 An actor-partner interdependence model of stigma and quality of life in schizophrenia patient-family caregiver dyads

This study revealed significant actor effects for both patient-caregiver dyad members, demonstrating that patient self-stigma and family caregiver affiliate stigma were inversely associated with their respective QoL. For individuals with schizophrenia, this association can be understood through the lens of stigma internalization. Patients with high levels of self-stigma often internalize negative societal and familial evaluations (such as prejudice and discrimination), leading to self-stigmatization (36). This internalized stigma is frequently accompanied by feelings of inferiority, which are closely correlated with lower self-esteem, social withdrawal, and illness concealment (7). These experiences, in turn, are commonly linked to heightened loneliness and helplessness, factors that prior research has associated with reduced social support-seeking and lower treatment adherence (7). For family caregivers, the actor effect highlights the profound impact of affiliate stigma—the perceived social discrimination and exclusion resulting from their association with the patient (8). Fearing public judgment, caregivers often report restricted social interactions and attempts to conceal the illness, leading to a state of ‘shared isolation’ with the patient that is positively correlated with heavier objective and subjective burdens. During the long-term caregiving process, social isolation, financial strain, and persistent concerns about the patient’s prognosis frequently co-occur with anxiety and depressive symptoms, ultimately aligning with their compromised QoL (37).

Second, regarding partner effects, we observed significant cross-partner associations, demonstrating that the stigma reported by one dyad member was inversely correlated with the QoL of the other. This cross-sectional pattern aligns with prior dyadic research (8, 38). For individuals with schizophrenia, behaviors linked to high self-stigma—such as treatment non-adherence or social withdrawal—frequently co-occur with symptomatic worsening, which is a known correlate of increased caregiver burden (39). Concurrently, a patient’s self-isolation and negative emotions often co-exist with heightened psychological distress, helplessness, and anxiety in family caregivers, constituting an environment linked to lower caregiver QoL (40). Conversely, caregivers’ affiliate stigma was significantly associated with patients’ lower QoL. According to the literature, higher affiliate stigma may manifest alongside negative emotions and behaviors in caregivers—such as anxiety, irritability, over-control, or criticism—which are strongly correlated with increased psychological stress and lower QoL in patients (32). Furthermore, within traditional Chinese culture, which heavily emphasizes ‘saving face,’ caregivers experiencing high affiliate stigma may resort to overprotecting patients, restricting their social outings, or displaying distant attitudes to preserve family reputation. Such family dynamics have been shown to correlate negatively with patients’ confidence in recovery and their overall QoL (8). Taken together, these findings suggest that the stigma experiences and QoL of patients and caregivers are deeply intertwined, highlighting the necessity of viewing them as an interdependent dyad in future clinical research and dyadic interventions.

### 4.4 Clinical implications of actor and partner effects in patients with schizophrenia and their caregivers

Our study, using the APIM, demonstrates that stigma is significantly associated with QoL both within individuals (actor effects) and across partners (partner effects) in these dyads. Rather than confirming a causal sequence, these cross-sectional associations align with a theoretical framework suggesting an intertwined pattern of stigma within families of patients with schizophrenia. Intriguingly, the caregiver’s actor effect was numerically larger than that of the patient, and the partner effect originating from the caregiver demonstrated a substantial cross-person association. Specifically, the caregiver’s affiliate stigma was associated with lower patient QoL to a degree nearly comparable to the patient’s own self-stigma (38), highlighting the centrality of the caregiver’s experience. Consequently, caregivers’ affiliate stigma appears to be a pivotal correlate within this family unit, closely tied not only to their own distress but also to the patient’s well-being (8). These findings suggest that when designing family-based support programs, caregivers warrant consideration as a central focus. Future longitudinal and intervention studies are needed to test whether alleviating caregivers’ affiliate stigma could be associated with improvements in their own QoL (given the strong actor effect) and potentially mitigate its negative associations with the patient’s QoL (given the partner effect). If confirmed, caregiver-focused strategies could represent a valuable entry point for supporting the entire family system.

In conclusion, this study provides an in-depth analysis of the interdependent associations between stigma and the QoL of patients with schizophrenia and their family caregivers. Our findings demonstrate that stigma is prevalent in both groups and is negatively correlated with their QoL, characterized by robust actor and partner effects. Consequently, family-centered strategies that address dual de-stigmatization concurrently—supporting both patients’ self-stigma and caregivers’ affiliate stigma—represent crucial components of support systems aimed at promoting better QoL, recovery, and social integration.

Furthermore, while our study captures a cross-sectional snapshot of this dyadic relationship, longitudinal evidence suggests that these stigma-related constructs are highly dynamic. For instance, recent research on individuals with severe mental illness and their family caregivers receiving community-based psychosocial interventions has demonstrated that internalized stigma, family affiliate stigma, and caregiver burden can fluctuate over time and are highly amenable to targeted support (41). This implies that the actor and partner effects observed in our study may also evolve across different stages of the illness and treatment, highlighting the critical importance of implementing long-term, community-based psychosocial interventions to mitigate the enduring interpersonal impact of stigma within patient-caregiver dyads. Ultimately, future longitudinal and experimental research is essential to elucidate the temporal relationships and directional pathways of these associations, thereby informing the efficacy of targeted, dyadic interventions for individuals with schizophrenia and their families.

### 4.5 Limitations

This study has several limitations. First, the cross-sectional design, while revealing significant associations between stigma and quality of life—particularly the actor and partner effects identified through the APIM—precludes the determination of causality. Second, the limited sample source (i.e., inpatients from Northern China) may affect the generalizability of our findings to the broader population of individuals with schizophrenia and their caregivers. Furthermore, three important methodological limitations should be noted. The first is the omission of key clinical and contextual covariates (e.g., symptom severity, caregiving burden), which may have confounded the observed associations. The second is the presence of high correlations among some variables, which suggests potential multicollinearity and calls for a cautious interpretation of the path estimates. Interestingly, the high correlation among caregiver variables might also reflect the authentic, ‘fused’ experience of their stigma and quality of life, a finding that warrants further investigation in itself. The third limitation is the reliance on self-report measures for stigma and quality of life, which could be susceptible to biases such as social desirability or recall bias. Therefore, future research should employ longitudinal designs, include more diverse samples, and integrate multi-source data with key covariates. Additionally, efforts should be made to mitigate the impact of multicollinearity among variables to build more robust models that can validate and extend our findings.

## 5 Conclusions

This study constructed an Actor-Partner Interdependence Model (APIM) to systematically investigate the impact of stigma on the Quality of Life (QoL) of patients with schizophrenia and their family caregivers. The findings indicate that stigma is prevalent in both groups and not only negatively affects an individual’s own QoL (an actor effect) but also significantly impacts their partner’s QoL (a partner effect). Consequently, it is crucial for interventions and support systems in schizophrenia care to adopt comprehensive strategies that concurrently address the issue of stigma in both patients and family caregivers, as this is a key pathway to improving their QoL. This research not only enriches the understanding of the impact of mental illness stigma but also provides new perspectives and insights for future clinical practice, emphasizing the importance of a family-centered, dual de-stigmatization model for promoting patient recovery and improving family functioning.

## Data Availability

The raw data supporting the conclusions of this article are not publicly available. Requests to access the datasets should be directed to Kang Xie, 839369050@qq.com.
